# Supplementary material for: Resources available for autism research in the big data era: a systematic review
Source: PeerJ. 2017 Jan 12;5:e2880. doi: 10.7717/peerj.2880 (PMC5237363; doi:10.7717/peerj.2880)
Supplement: Supplemental Information 2 [file peerj-05-2880-s003.docx]

The rationale for conducting a systematic review / search on this area was to provide the ASD research community with a comprehensive and detailed description of ASD resources of multiple data-types that can be accessed, shared, and analysed by independent researchers regardless of funding source; and thus this would likely lead to multi-level or large scale impactful research. The review is also in response to the evident move towards generating ‘big’ and ‘open’ data in many areas of science such as psychology, neuroscience, genetics, and omics.

The value of big, open data research is increasingly being recognized by many stakeholders involved in the academic research process, including funding bodies, publishers, researchers, and participants. It is regarded as an important step forward, especially in research relating to complex heterogeneous disorders such as ASD where the inconsistencies of findings and the great heterogeneity of the disorder necessitate the use of big and open data. To this end, a number of initiatives have been established that aim to develop big and / or open data resources for autism research. Consequently, several existing reviews have accumulated a general list of available data resources, although these are not ASD-specific [1-5]. Although useful, these lists are non-comprehensive and are mainly limited to general neuroimaging resources. In addition, some of the lists included resources that are inaccessible to researchers (e.g. limited access data to only respective consortia members), and thus would not be beneficial to the research community at large. Most importantly, to date, no existing article provides a comprehensive list and analysis of available sources of ASD data despite the growing interest and need in generating big / open data for ASD research.

A number of authors [1-5] discuss the potential importance of big and / or open data for the progression of research and the factors that encouraged the application of this philosophy. The submitted paper further adds to this discussion by highlighting the importance of secondary shared data (big and / or open data), specifically, for research relating to complex heterogeneous disorders such as ASD and the study of potential ASD subtypes.

# References

1. Van Horn JD, Scott TG, Rockmore D, Michael SG. Sharing neuroimaging studies of human cognition. Nat Neurosci. 2004;7(5):473.
2. Van Horn JD, Wolfe J, Agnoli A, Woodward J, Schmitt M, Dobson J, et al. Neuroimaging databases as a resource for scientific discovery. Int Rev Neurobiol. 2005;66:55-87.
3. Van Horn JD, Toga AW. Is it time to re- prioritize neuroimaging databases and digital repositories? Neuroimage. 2009;47(4):1720-1734.
4. Ferguson AR, Nielson JL, Cragin MH, Bandrowski AE, Martone ME. Big data from small data: data- sharing in the ' long tail' of neuroscience. Nat Neurosci. 2014;17(11):1442-1447.
5. Poldrack R, Gorgolewski K. Making big data open: data sharing in neuroimaging. Nat Neurosci. 2014;17(11):1510-1517.
